# Efficacy and Safety of Oral Herbal Medicine Combined with Diosmectite for Pediatric Rotavirus Gastroenteritis: A Systematic Review and Meta-Analysis

**DOI:** 10.3390/healthcare14060711

**Published:** 2026-03-11

**Authors:** Jung-Hyun Cho, Eun-Jin Kim, Hyun-Kyung Sung, Sang-Yeon Min

**Affiliations:** 1 Department of Pediatrics of Korean Medicine, Graduate School of Dongguk University, Seoul 04620, Republic of Korea; cphantom@naver.com; 2 Baruni Oriental Medicine Clinic, Suwon-si 16703, Gyeonggi-do, Republic of Korea; 3 Department of Pediatrics of Korean Medicine, Korean Medicine Hospital, Dongguk University Bundang Medical Center, Seongnam-si 13601, Gyeonggi-do, Republic of Korea; utopialimpid@naver.com; 4 Department of Education, College of Korean Medicine, Dongguk University, Gyeongju-si 38066, Gyeongsangbuk-do, Republic of Korea; shksolar@gmail.com; 5 Department of Pediatrics of Korean Medicine, Korean Medicine Hospital, Dongguk University Ilsan Medical Center, Goyang-si 10326, Gyeonggi-do, Republic of Korea

**Keywords:** herbal medicine, rotavirus gastroenteritis, diosmectite, complementary treatment, meta-analysis

## Abstract

**Background/Objectives**: Rotavirus is a leading cause of severe acute gastroenteritis in children and is commonly associated with prolonged diarrhea and dehydration. Herbal medicine (HM) is frequently used in combination with diosmectite in clinical practice, but the effectiveness of this combined approach has not been systematically evaluated. This study aimed to assess the efficacy and safety of HM combined with diosmectite in pediatric rotavirus gastroenteritis. **Methods**: We searched 12 databases from inception to 10 December 2025. Randomized controlled trials comparing HM plus diosmectite with diosmectite alone in children with rotavirus gastroenteritis were included. Study selection, data extraction, and risk-of-bias assessment were independently performed by two researchers. Meta-analyses were conducted using RevMan 5.4, and the certainty of evidence was evaluated with GRADEpro. **Results**: A total of 26 RCTs involving 2876 children were included. Compared with diosmectite alone, HM combined with diosmectite significantly reduced the duration of diarrhea (SMD −1.31; 95% CI −1.63 to −0.99), improved the total effective rate (RR 1.25; 95% CI 1.19 to 1.31), decreased the incidence of adverse events (RR 0.24; 95% CI 0.06 to 0.92), and a shorter length of hospital stay (MD −1.53; 95% CI −1.73 to −1.33). The certainty of evidence ranged from moderate to very low. **Conclusions**: HM combined with diosmectite may offer a potential adjunctive therapy for pediatric rotavirus gastroenteritis. However, more robust and high-quality evidence is required to further substantiate its efficacy and safety.

## 1. Introduction

Rotavirus is the leading cause of severe acute gastroenteritis in children under five years of age and remains the predominant pathogen responsible for diarrheal mortality in this age group [1,2]. The incidence of the clinical disease peaks between 4 and 23 months of age, the period during which children are at the highest risk of developing severe illnesses requiring hospitalization [3]. The clinical manifestations of rotavirus infection vary widely, ranging from mild to severe vomiting and watery diarrhea, which can lead to dehydration, electrolyte imbalance, shock, and even death if appropriate fluid replacement is not provided. Although gastrointestinal symptoms usually resolve within 3–7 days and may persist for 2–3 weeks, there is no specific antiviral therapy for rotavirus infection [4]. Consequently, the cornerstone of clinical management is the prevention and treatment of dehydration, with timely and adequate rehydration being the most critical therapeutic goal.

Diosmectite, also known as dioctahedral smectite, is commonly used as an adjunctive therapy in clinical practice alongside oral rehydration therapy. According to a Cochrane review, diosmectite may modestly reduce the duration of diarrhea, increase the rate of clinical resolution by day 3, and decrease stool output in children with acute gastroenteritis [5]. It is a natural clay composed of layered aluminum and magnesium silicates that act locally in the intestinal lumen without systemic absorption. Unlike antidiarrheal agents, such as loperamide or racecadotril, it exerts its effects through physicochemical and barrier-protective mechanisms rather than by altering intestinal motility or secretion.

In real-world clinical practice, Herbal medicine (HM) is frequently administered alongside conventional treatments, including diosmectite, to enhance symptom control and recovery. While previous reviews have assessed the effects of HM in pediatric rotavirus gastroenteritis [6,7,8], the effects of combining HM with diosmectite have not been systematically evaluated. It therefore remains uncertain whether this combination offers additional clinical advantages over diosmectite alone or standard supportive care. Accordingly, we conducted a systematic review and meta-analysis to assess the effectiveness and safety of HM combined with diosmectite in children with rotavirus gastroenteritis.

## 2. Materials and Methods

### 2.1. Protocol and Registration

This systematic review and meta-analysis were conducted in accordance with the Preferred Reporting Items for Systematic Reviews and Meta-Analyses (PRISMA) guidelines [9] (Supplementary Table S1). The review protocol was registered in PROSPERO (ID: CRD420251271002) prior to conducting the review and is available at: https://www.crd.york.ac.uk/PROSPERO/view/CRD420251271002 (accessed on 21 December 2025).

### 2.2. Eligibility Criteria

#### 2.2.1. Types of Studies

Only randomized controlled trials (RCTs) evaluating the combination of oral HM and diosmectite for the treatment of pediatric rotavirus enteritis were included. Trials using quasi-random allocation methods such as admission order, alternation, date of birth, and number of medical records were excluded. Observational studies, animal experiments, case reports or case series, surveys, gray literature, theses and dissertations, and narrative or systematic reviews were not considered eligible.

#### 2.2.2. Types of Participants

Participants were pediatric patients under 18 years of age who had been diagnosed with rotavirus enteritis according to established clinical or laboratory diagnostic criteria. Participants were excluded if they had infectious diarrhea caused by pathogens other than rotavirus or had systemic diseases or comorbid conditions that could affect disease progression or treatment response, including immunodeficiency disorders, severe malnutrition, gastrointestinal vascular malformations, or congenital heart disease.

#### 2.2.3. Types of Interventions

The experimental group was administered HM in combination with diosmectite. Only orally administered HM was permitted, with no restrictions on the number of constituent herbs, formulation type (including powders, pills, granules, capsules, decoctions, and oral solutions), dosage, or treatment duration. Participants in the experimental group received standard treatment for rotavirus enteritis, provided that the same treatment was administered to the control group.

#### 2.2.4. Types of Comparisons

The control group received diosmectite and was permitted standard treatment for rotavirus enteritis, including fluid and electrolyte replacement and symptomatic supportive care, such as treatment for vomiting and fever, when necessary. To minimize clinical heterogeneity, trials were excluded if additional pharmacological treatments such as antibiotics, digestive agents, antiviral agents, probiotics, and zinc supplementation were administered.

#### 2.2.5. Types of Outcome Measurements

The primary outcomes focused on the resolution of diarrhea, including the duration of diarrhea, defined as the number of days from the initiation of treatment to the complete cessation of diarrheal symptoms, and the total effective rate (TER), calculated as the proportion of patients classified as recovered or improved, divided by the total number of patients. Secondary outcomes included the incidence of adverse events, length of hospital stay, and resolution of upper gastrointestinal symptoms, measured as the time to the cessation of vomiting and fever. Additionally, changes in immune and inflammatory markers, including CRP, IL-6, and IL-8, were evaluated.

### 2.3. Information Sources and Search Strategy

Twelve electronic databases were searched without restrictions on language or year of publication until 10 December 2025. Twelve electronic databases were searched without restrictions on language or year of publication until 10 December 2025. The English databases included MEDLINE (via PubMed), Embase (via Elsevier), and the Cochrane Central Register of Controlled Trials. In addition, regional databases were searched, including three Chinese databases (China National Knowledge Infrastructure, Wanfang Data, and the Chinese Scientific Journal Database), five Korean medical databases (Korea Citation Index, Korean Medical Database, Korean Studies Information Service System, Oriental Medicine Advanced Searching Integrated System, and Research Information Sharing Service), and the Japanese database Citation Information by Nii. The search strategy incorporated terms related to pediatric populations, rotavirus enteritis or diarrhea, and herbal medicine. The search terms were modified as appropriate for each database. Detailed search strategies and corresponding results for each database are provided in Supplementary Table S2.

### 2.4. Study Selection and Data Extraction

#### 2.4.1. Study Selection

Two researchers (J.-H.C. and E.-J.K.) independently reviewed the titles and abstracts of all retrieved records and evaluated the full texts of potentially eligible studies in accordance with the predefined inclusion and exclusion criteria using EndNote software (version 20, Clarivate Analytics, Philadelphia, PA, USA). The researchers independently completed the selection procedure and compared the assessments. Any disagreements were resolved by consensus through discussion, and unresolved discrepancies were adjudicated by a third researcher (S.-Y.M.).

#### 2.4.2. Data Extraction

Data were independently extracted by two researchers (J.-H.C. and E.-J.K.). In cases of discrepancies during data extraction, all authors discussed issues to reach a consensus. If essential data were missing or unclear, we attempted to obtain clarification by contacting the study authors via email. Data extracted from each eligible study included bibliographic information (first author and year of publication), sample size, participant characteristics (age, sex distribution, and duration of illness), intervention characteristics (treatment duration and details of the intervention and comparator), outcome measures, comparative results, reported adverse events, and information required for risk of bias (RoB) assessment.

### 2.5. Assessment of RoB

Methodological quality was evaluated independently by two researchers (J.-H.C. and E.-J.K.) using the Cochrane RoB 2 tool. Each study was classified as having a low risk of bias, some concerns, or a high risk of bias [10]. The tool assesses potential bias across five domains: the randomization process, deviations from intended interventions, missing outcome data, outcome measurement, and selective reporting. Based on the judgments across these domains, the overall risk of bias was determined for each study. Discrepancies between researchers were resolved through discussion, and when consensus could not be reached, a third researcher (S.-Y.M.) participated in the final decision.

### 2.6. Statistical Analysis

All included studies were synthesized qualitatively. When at least two studies reported comparable continuous or dichotomous outcomes, a meta-analysis was conducted using Review Manager (version 5.4.1; Cochrane Collaboration, Copenhagen, Denmark). For dichotomous outcomes, effect estimates were expressed as risk ratios (RR) with 95% confidence intervals (CI). For continuous outcomes, mean differences (MD) or standardized mean differences (SMD) with 95% CIs were calculated. SMD were applied when the same outcome was assessed using different measurement scales or units across the studies. Statistical heterogeneity was evaluated using the I^2^ statistic [11]. An I^2^ value exceeding 50% was considered indicative of substantial heterogeneity, and a random-effects model was therefore applied. When the I^2^ value was 50% or lower, a fixed-effects model was used.

#### 2.6.1. Assessment of Reporting Bias

Publication bias was investigated when at least 10 studies were available. Funnel plots were generated to assess possible asymmetry, and Egger’s regression test [12] was conducted to statistically evaluate the presence of small-study effects. When a publication bias was suspected, the robustness of the pooled estimates was further examined using Rosenthal’s fail-safe N [13] and the trim-and-fill method [14]. All publication bias analyses were performed using R software (version 4.1.1; R Foundation for Statistical Computing, Vienna, Austria) and R Studio (version 1.4.1106; RStudio, PBC, Boston, MA, USA), employing meta and metafor packages with default settings.

#### 2.6.2. Subgroup and Sensitivity Analysis

Given the diversity in HM composition, subgroup analyses were planned according to specific HM types when a sufficient number of studies were available. Sensitivity analyses were performed for outcomes reported in at least 10 studies using a leave-one-out approach, in which each study was sequentially excluded to evaluate the robustness of the pooled estimates.

### 2.7. Quality of Evidence

The certainty of evidence was evaluated using the Grading of Recommendations Assessment, Development, and Evaluation (GRADE) approach with the GRADEpro tool (http://gradepro.org). The evidence was assessed across five domains: risk of bias, inconsistency of results, indirectness of evidence, imprecision of effect estimates, and publication bias. Based on these assessments, the overall certainty was rated as high, moderate, low, or very low [15].

## 3. Results

### 3.1. Study Selection

**Figure 1:**
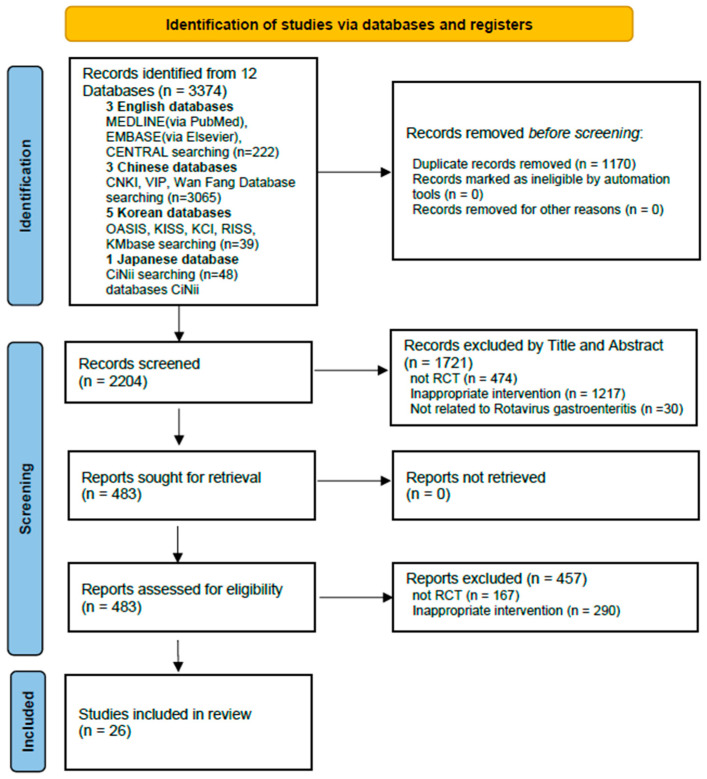
PRISMA flow diagram of study selection process. MEDLINE, Medical Literature Analysis and Retrieval System Online; EMBASE, Excerpta Medica database; CENTRAL, Cochrane Central Register of Controlled Trials; CNKI, China National Knowledge Infrastructure; VIP, Chinese Scientific Journal Database; OASIS, Oriental Medicine Advanced Searching Integrated System; KISS, Korean studies Information Service System; KCI, Korea Citation Index; RISS, Research Information Sharing Service; CiNii, Citation Information by NII; RCT, randomized controlled trial.

After applying the search strategy across all databases, 3374 records were identified, including 222 from English, 3065 from Chinese, 39 from Korean, and 48 from Japanese databases. After removing duplicates, 2204 records remained for screening. Title and abstract screening resulted in the selection of 483 articles for full-text assessment. Of these, 167 studies were excluded because they were not RCTs, and 290 studies were excluded because of inappropriate interventions. Finally, 26 RCTs [16,17,18,19,20,21,22,23,24,25,26,27,28,29,30,31,32,33,34,35,36,37,38,39,40,41] were included in the systematic review and meta-analysis (Figure 1).

### 3.2. Characteristics of the Study

All 26 included studies were RCTs conducted in China and published between 2004 and 2025. The sample size ranged from 64 to 312 participants, and the treatment duration varied from three days to two weeks. Participants’ ages ranged from two months to seven years, with most studies enrolling children younger than three years. The baseline disease duration ranged from approximately 1 to 4 days, and most participants had a symptom duration of less than 3 days. The detailed study characteristics are presented in Table 1.

### 3.3. Interventions

Across the 26 included trials, oral HM was used as an intervention. These included classical herbal prescriptions and their modified forms, Chinese patent medicines, self-formulated decoctions, pediatric-specific preparations, and syndrome-differentiated prescriptions (Table 2).

Classical herbal prescriptions and modified formulas included the Gegen Qinlian decoction [18,21,27,31], Qiwei Baizhu powder [19,37], Zhixie Weiling decoction [26,33], Shenling Baizhu powder combined with the Wuji pill [38], Zhenren Yangzang decoction [28], and modified Yigong san [20]. Patented Chinese medicines include Fengliao Changweikang granules [24,34], Weichang an pill [16], compound Ocimum oil oral liquid [36], and Shuanghuanglian oral liquid [39]. Self-formulated decoctions have been reported in several trials, including Zhixie decoction [25], Jianpi Qushi decoction [32], and Qiuxie decoction [40], which are used for the management of gastroenteritis-related symptoms. Pediatric-specific patent medicines are commonly used, including Xiaoer tuxiening powder [29], Erxieting granules [30,35], Xiaoer shikou powder [17], Xiaoer zhixie granules [22], and Xingpi Yanger granules [41], all of which are traditionally prescribed for gastrointestinal disorders in children. In addition, one study [23] employed syndrome-differentiated prescriptions in which herbal formulas were tailored according to individual pattern differentiation based on traditional HM theory. These included the Baohe pill for food retention-induced diarrhea, Huoxiang Zhengqi powder for wind-cold diarrhea, Gegen Qinlian decoction for damp-heat diarrhea, Shenling Baizhu powder for spleen-deficiency diarrhea, and warming and tonifying therapies for spleen–kidney yang deficiency.

The most frequently used herbal components across the included trials were Glycyr-rhizae Radix et Rhizoma (17 occurrences), Poria (13 occurrences), Puerariae Radix and Atractylodis Macrocephalae Rhizoma (11 occurrences each), followed by Coptidis Rhi-zoma and Scutellariae Radix (7 occurrences each). Detailed information is provided in Supplementary Table S3. All studies administered diosmectite to the experimental and control groups with additional standard supportive care, such as fluid therapy, oral rehydration salts, and dietary guidance (Supplementary Table S4).

### 3.4. Outcome Measures

The primary outcomes were the resolution of diarrhea, including the duration of diarrhea and the TER. Fourteen studies reported the duration of diarrhea [16,17,18,19,21,23,25,27,29,31,34,37,39,41]. However, two studies [23,29] did not provide sufficient quantitative data for the meta-analysis and were therefore excluded from the pooled analysis of this outcome. The TER was reported in 25 studies [16,17,18,19,20,21,22,23,24,25,26,27,28,29,30,31,32,33,34,35,36,37,38,39,40], all of which were included in the corresponding meta-analysis.

The secondary outcomes included the incidence of adverse events [22,28,29,32,35,36,42]. However, one study [29] did not report quantitative data on event frequency and was therefore excluded from the meta-analysis of adverse event incidence. Other secondary outcomes included the length of hospital stay [18,36,39] and resolution of upper gastrointestinal symptoms, assessed as the time to cessation of vomiting [16,17,18,21,27,31,37,39,41] and fever [16,17,18,21,25,27,31,36,37,41]. One study [40] reported vomiting and fever outcomes only as effective rates and was therefore excluded from the quantitative analysis of symptom resolution. In addition, changes in the levels of inflammatory biomarkers, including CRP [17,41], IL-6 [17,27,41], and IL-8 [17,41] were evaluated. Detailed results for all outcome measures, including their corresponding p-values, are presented in Supplementary Table S5.

### 3.5. Quality Assessment

Regarding bias arising from the randomization process, all studies stated that random allocation was performed; however, none provided sufficient information on whether allocation concealment was ensured during participant enrollment and assignment to interventions, resulting in concerns across studies. Two studies [23,29] were judged to be at high risk of bias owing to missing outcome data because of incomplete outcome reporting. For bias in the measurement of the outcome, studies that assessed only TER as the outcome measure [20,24,26,29,30,33,38] were judged to be at a high risk of bias, as this outcome relies on subjective assessment and lacks standardized, objective measurement criteria.

**Figure 2:**
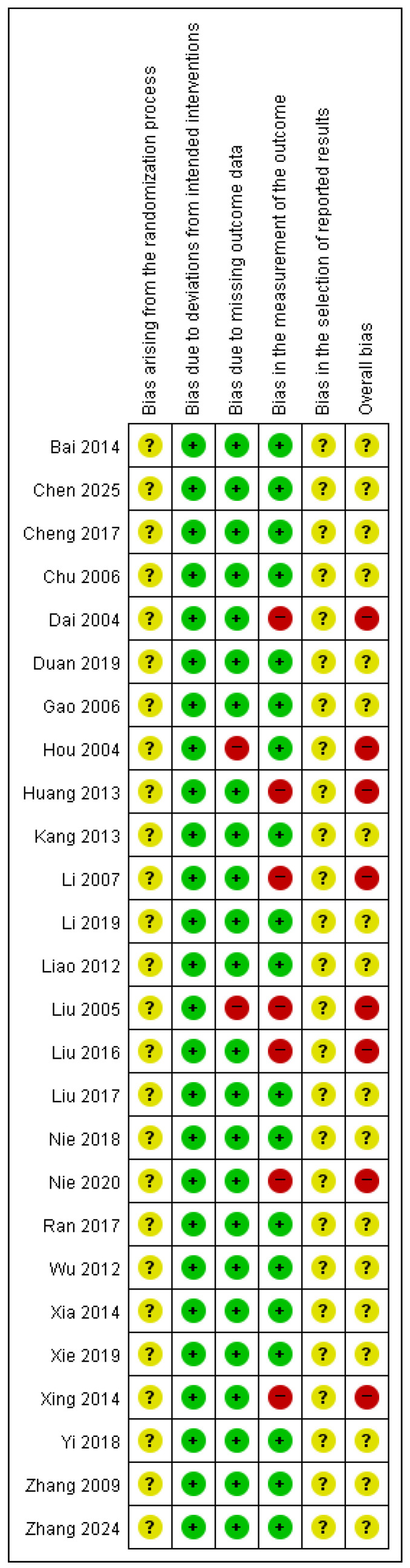
Risk of bias assessment [16,17,18,19,20,21,22,23,24,25,26,27,28,29,30,31,32,33,34,35,36,37,38,39,40,41]. +, Low risk of bias; ?, Unclear risk of bias; −, High risk.

**Figure 3:**
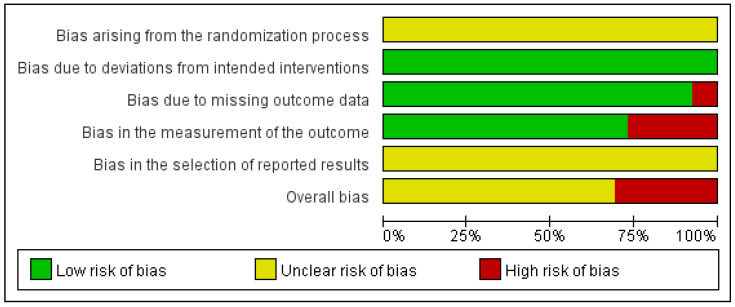
Risk of bias graph.

Consequently, eight studies [20,23,24,26,29,30,33,38] were categorized as having a high risk of bias, while the remaining studies were rated as having some concerns. The overall risk of bias assessment is shown in Figure 2 and Figure 3.

### 3.6. Meta-Analysis Results

A meta-analysis was conducted to compare the efficacy of HM combined with diosmectite with that of diosmectite alone in the management of pediatric rotavirus gastroenteritis.

#### 3.6.1. Duration of Diarrhea (Days)

**Figure 4:**
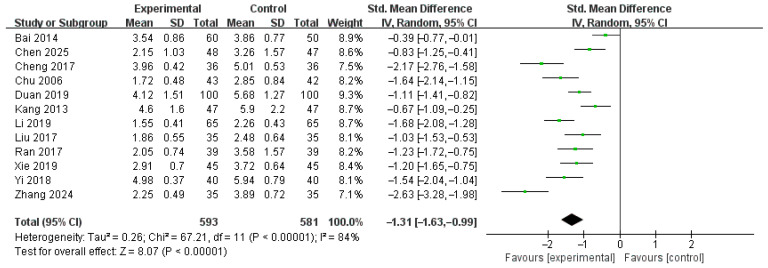
Forest plot of the duration of diarrhea [16,17,18,19,21,25,27,31,34,37,39,41].

Twelve studies [16,17,18,19,21,25,27,31,34,37,39,41], involving 1174 patients, were included in the meta-analysis. In two studies [19,31], the duration of diarrhea was assessed in hours and subsequently converted to days to ensure comparability across studies; therefore, the analysis was conducted using SMD. Under a random-effects model, the pooled SMD was −1.31 days (95% CI: −1.63 to −0.99), suggesting a reduction in diarrhea duration when HM was used as an adjunct to diosmectite, relative to diosmectite alone (Figure 4). Although considerable heterogeneity was observed across the studies (I^2^ = 84%, *p* < 0.00001), the overall effect remained statistically significant (Z = 8.07, *p* < 0.00001), suggesting that HM may be effective in shortening the duration of diarrhea in pediatric patients with rotavirus gastroenteritis.

#### 3.6.2. TER

**Figure 5:**
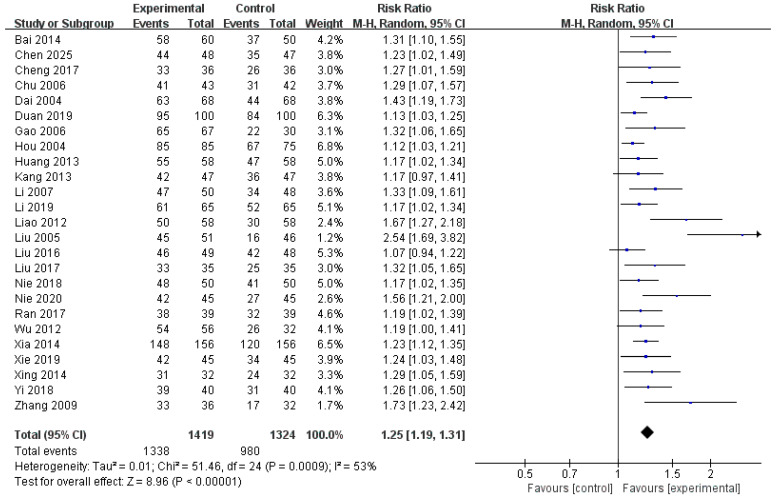
Forest plot of the total effective rate [16,17,18,19,20,21,22,23,24,25,26,27,28,29,30,31,32,33,34,35,36,37,38,39,40].

A total of 25 studies [16,17,18,19,20,21,22,23,24,25,26,27,28,29,30,31,32,33,34,35,36,37,38,39,40] involving 2743 pediatric patients assessed the TER of HM combined with diosmectite compared with that of diosmectite alone. The pooled analysis yielded an RR of 1.25 (95% CI: 1.19 to 1.31), indicating that the experimental group achieved a significantly higher rate of clinical improvement than the control group (Figure 5). Moderate heterogeneity was observed across studies (I^2^ = 53%, *p* = 0.0009). Therefore, a random-effects model was used. The overall effect was highly statistically significant (Z = 8.96, *p* < 0.00001), supporting the consistent and favorable effect of HM combined with diosmectite compared with that of diosmectite alone in the treatment of pediatric rotavirus gastroenteritis.

#### 3.6.3. Incidence of Adverse Events

**Figure 6:**
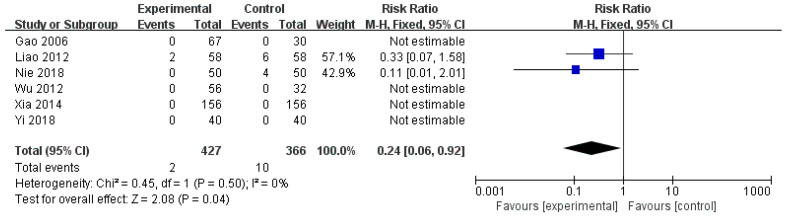
Forest plot of adverse events [22,28,32,35,36,39].

Six studies [22,28,32,35,36,39], involving 793 participants, reported adverse events related to the combination of HM and diosmectite. Pooled analysis demonstrated a significantly reduced risk of adverse events in the experimental group compared with the control group (RR = 0.24, 95% CI: 0.06 to 0.92; Figure 6). No statistical heterogeneity was detected across the included studies (I^2^ = 0%, *p* = 0.50). Therefore, we use a fixed-effects model in this study. The overall effect was statistically significant (Z = 2.08, *p* = 0.04), suggesting that the addition of HM to diosmectite did not increase adverse events and may be associated with a favorable safety profile for the treatment of pediatric rotavirus gastroenteritis.

#### 3.6.4. Length of Hospital Stay (Days)

**Figure 7:**
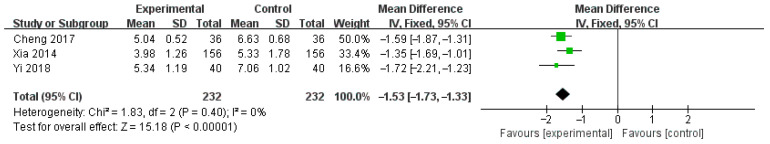
Forest plot of length of hospital stay [18,36,39].

Three studies [18,36,39], involving 464 patients, assessed length of hospital stay. The pooled analysis showed that HM combined with diosmectite significantly shortened the duration of hospitalization compared with diosmectite alone (MD = −1.53, 95% CI: −1.73 to −1.33, Figure 7). No heterogeneity was observed among the included studies (I^2^ = 0%) and a fixed-effects model was used. The overall effect was statistically significant (Z = 15.18, *p* < 0.00001).

#### 3.6.5. Resolution of Upper Gastrointestinal Symptoms (Days)

**Figure 8:**
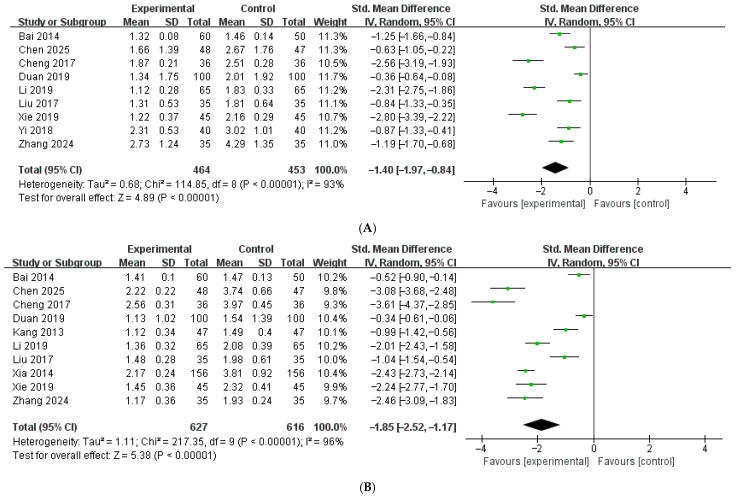
(**A**) Forest plot of duration of vomiting [16,17,18,21,27,31,37,39,41]. (**B**) Forest plot of duration of fever [16,17,18,21,25,27,31,36,37,40].

Nine studies [16,17,18,21,27,31,37,39,41], enrolling 917 patients, evaluated the duration of vomiting, defined as the time to resolution of vomiting symptoms. One study [31] reported symptom duration in hours, which was converted to days for analysis. The meta-analysis demonstrated a significant pooled effect, with HM combined with diosmectite significantly reducing the duration of vomiting compared with diosmectite alone (SMD = −1.40, 95% CI: −1.97 to −0.84, Figure 8A). Substantial heterogeneity was observed across studies (I^2^ = 93%, *p* < 0.00001). The overall effect was statistically significant (Z = 4.89, *p* < 0.00001). Ten studies [16,17,18,21,25,27,31,36,37,40], enrolling 1243 patients, evaluated the duration of fever, defined as the time to resolution of fever symptoms. In two studies [25,31], symptom duration was reported in hours and subsequently converted to days to ensure consistency across studies. The meta-analysis demonstrated a significant pooled effect, with HM combined with diosmectite significantly reducing the duration of fever compared with diosmectite alone (SMD = −1.85, 95% CI: −2.52 to −1.17, Figure 8B). Substantial heterogeneity was observed across studies (I^2^ = 96%, *p* < 0.00001). The overall effect was statistically significant (Z = 5.38, *p* < 0.00001).

#### 3.6.6. Changes in Inflammatory Biomarkers

**Figure 9:**
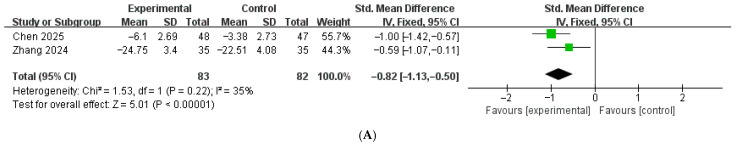

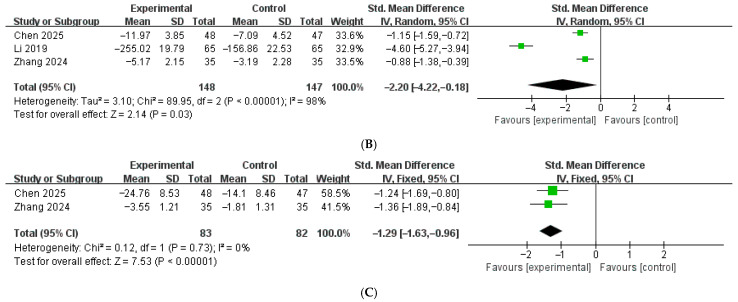
(**A**) Forest plot of CRP (mg/L) [17,41]. (**B**) Forest plot of IL-6 (pg/mL) [17,27,41]. (**C**) Forest plot of IL-8 (pg/mL) [17,41].

Two studies [17,41] involving 165 patients reported CRP levels before and after treatment, showing a significant reduction in CRP in the experimental group compared with the control group (SMD = −0.82, 95% CI: −1.13 to −0.50, I^2^ = 35%, *p* = 0.22, Figure 9A). Three studies [17,27,41] involving 295 patients reported IL-6 levels, with the pooled analysis demonstrating a significant reduction (SMD = −2.20, 95% CI: −4.22 to −0.18, Figure 9B), although substantial heterogeneity was observed (I^2^ = 98%, *p* < 0.00001). In addition, two studies [17,41] involving 165 patients reported IL-8 levels, and the pooled analysis showed a significant reduction (SMD = −1.29, 95% CI: −1.63 to −0.96, I^2^ = 0%, *p* = 0.73, Figure 9C).

### 3.7. Assessment of Reporting Bias

**Figure 10:**
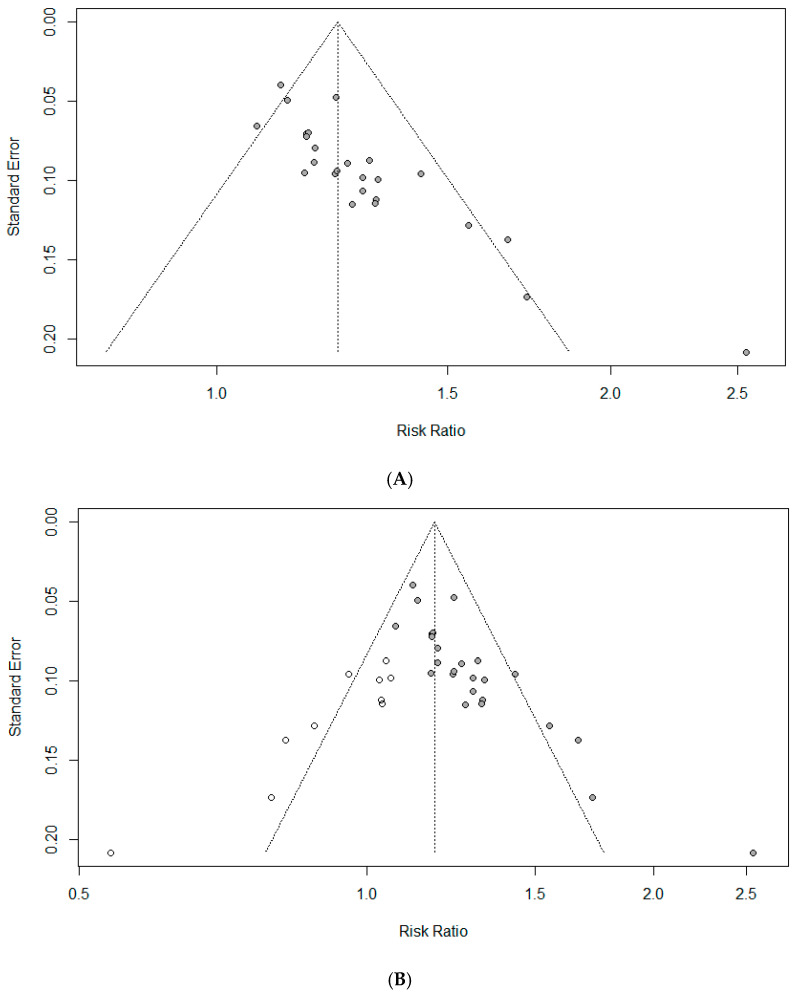
(**A**) Funnel plot of the TER. (**B**) Trim and fill adjusted funnel plot of the TER. The closed dots represent real studies and the open dots represent filled artificial studies.

Graphical and statistical methods were used to assess the potential impact of publication bias. The funnel plot for TER indicated a potential publication bias (Figure 10A), which was further corroborated by Egger’s regression test, demonstrating a statistically significant funnel plot asymmetry (*p* < 0.001), suggesting the presence of small-study effects. To evaluate the influence of this potential bias on the pooled estimate, the trim and fill method was applied, which resulted in the imputation of 10 potentially missing studies (Figure 10B). After adjustment, the pooled effect size was attenuated from the original estimate of RR = 1.24 (95% CI: 1.19–1.29) to RR = 1.18 (95% CI: 1.12–1.24) while remaining statistically significant, indicating that although publication bias may have contributed to some overestimation of the effect, the overall direction and statistical significance of the association were preserved. In addition, Rosenthal’s fail-safe N was estimated to be 1613, substantially exceeding the conventional threshold of 135 (calculated as 5k + 10, where k = 25 is the number of included RCTs), suggesting that an implausibly large number of unpublished null studies would be required to overturn the observed findings. Collectively, these results indicate that, although publication bias cannot be entirely ruled out, its influence on the conclusions of the present meta-analysis is likely limited, and the findings appear to be robust.

### 3.8. Subgroup and Sensitivity Analyses

Subgroup analyses based on specific HM formulations were not performed because the number of RCTs for each formulation was insufficient. Sensitivity analyses were performed to assess the robustness of the pooled results. When eight studies [20,23,24,26,29,30,33,38] assessed as having an overall high risk of bias were excluded, the pooled estimate for TER remained similar (RR 1.23; 95% CI 1.15–1.28), with low heterogeneity (I^2^ = 3%, *p* = 0.41), indicating that the primary finding was not materially influenced by high-risk studies. In addition, leave-one-out sensitivity analyses demonstrated that exclusion of any single study did not substantially alter the overall effect estimate (RR = 1.23–1.26, all *p* < 0.00001), and heterogeneity (I^2^ = 34–56%) remained moderate (Supplementary Table S6). These findings support the stability of the pooled results.

### 3.9. GRADE Certainty of Evidence

According to the GRADE assessment, the certainty of evidence differed across outcomes (Table 3). Significant improvements were observed in key clinical outcomes, including duration of diarrhea (SMD −1.31, 95% CI −1.63 to −0.99; 12 RCTs, n = 1174) and TER (RR 1.25, 95% CI 1.19 to 1.31; 25 RCTs, n = 2743). However, the certainty of evidence for these outcomes was rated as very low, mainly because of the methodological limitations of the included trials, substantial heterogeneity, and concerns regarding publication bias. In contrast, the evidence for safety and several secondary outcomes was more consistent. The incidence of adverse events was lower in the intervention group (RR 0.24, 95% CI 0.06 to 0.92; 6 RCTs, n = 793), with no observed heterogeneity, resulting in moderate certainty of evidence. Moderate-certainty evidence also supported reductions in CRP and IL-8 levels, as well as length of hospital stay. Evidence for IL-6 and symptom duration outcomes (vomiting and fever) was of low to very low certainty because of considerable heterogeneity and imprecision, despite statistically significant pooled effects. Overall, while the intervention was associated with favorable effects across multiple outcomes, the reliability of these findings remains limited by the quality and consistency of the available evidence.

## 4. Discussion

### 4.1. Summary of This Review

This systematic review and meta-analysis indicate that combining HM with diosmectite provides greater therapeutic benefits than diosmectite alone for pediatric rotavirus gastroenteritis. Subgroup analyses by HM type were not performed because the available data were insufficient and conducting such analyses could have compromised the quality and reliability of the results. A total of 26 RCTs involving 2876 children were included in this study. The results showed that combination therapy significantly reduced the duration of diarrhea and improved the TER, while also lowering the incidence of adverse events. Inflammatory markers, including CRP, IL-6, and IL-8, were also significantly reduced. In addition, the combination therapy shortened length of hospital stay, as well as the duration of vomiting and fever. Publication bias could be assessed for TER, suggesting that although publication bias cannot be entirely excluded, its impact on the overall conclusions is likely limited, and the results appear to be robust. Overall, these findings suggest that HM may serve as a valuable adjunctive treatment for children with rotavirus gastroenteritis by improving symptom resolution when combined with diosmectite therapy.

### 4.2. Clinical Implications, Limitations, and Suggestions

In rotavirus gastroenteritis, the primary therapeutic goal is to prevent dehydration by shortening the duration of diarrhea and improving the associated symptoms. HM has traditionally been used for the treatment of diarrhea, and among the prescriptions most frequently applied in the included studies, the Gegen Qinlian decoction is one of the most commonly used formulas. The Gegen Qinlian decoction is composed of Puerariae Lobatae Radix, Scutellariae Radix, Coptidis Rhizoma, and Glycyrrhizae Radix et Rhizoma Prae-parata cum Melle and has been widely used for diarrheal diseases [42]. Pharmacological studies have demonstrated multiple actions relevant to diarrhea, including anti-inflammatory effects, gut microbiota modulation, intestinal mucosal barrier protection, and intestinal immune response regulation [43]. In addition, a recent systematic review and meta-analysis reported that the Gegen Qinlian decoction combined with conventional Western medicine improved clinical outcomes in patients with infectious diarrhea [44]. Across the 26 studies, several herbs were used frequently, including Glycyrrhizae Radix, Poria cocos, Puerariae Radix, and Atractylodis Macrocephalae Rhizoma, all of which are traditionally recognized for their roles in regulating gastrointestinal function, reducing inflammation, and supporting intestinal barrier recovery. Glycyrrhizae Radix has demonstrated anti-inflammatory effects by reducing pro-inflammatory mediators such as IL-1β, IL-6, and TNF-α in vitro, suggesting a role in mitigating gut inflammation [45]. Poria cocos has been traditionally used to support gastrointestinal function and fluid balance, which may contribute to symptomatic relief in diarrheal conditions [46]. Puerariae Radix, a key component of the Gegen Qinlian Decoction, has been associated with the modulation of intestinal immune responses, protection of the mucosal barrier, and regulation of gut microbiota in experimental models of intestinal inflammation [47]. Atractylodis Macrocephalae Rhizoma contains bioactive components such as atractylenolides, which have been shown to modulate inflammatory signaling pathways and contribute to protective effects in the stomach and intestine [48].

Diosmectite adsorbs bacteria, viruses, toxins, and inflammatory mediators, while reinforcing the gut mucus barrier by binding to mucins and mucoproteins, thereby limiting toxin penetration and protecting the intestinal epithelium. These actions help preserve epithelial integrity, enhance ion absorption, reduce water loss, and restore barrier function, leading to an improvement in diarrheal symptoms [49,50]. There have been no clinically significant food interactions reported. However, due to its adsorptive properties, appropriate time intervals between diosmectite and other orally administered medications are generally recommended. HM has been reported to modulate inflammatory responses, regulate gut microbiota, and promote intestinal barrier recovery. Taken together, diosmectite and HM may exert complementary effects, with diosmectite primarily limiting luminal injury and HM supporting inflammatory regulation and mucosal repair. However, as the precise interactions and synergistic mechanisms between these therapies have not been clearly elucidated, these interpretations should be made with caution.

Our meta-analysis had several important strengths. First, a comprehensive and robust literature search was conducted to identify relevant RCTs. To ensure methodological rigor, trials using quasi-random allocation methods such as assignment by admission order, alteration, date of birth, or number of medical records were excluded. Studies in which additional pharmacological treatments, including antibiotics, digestive agents, antiviral drugs, probiotics, or zinc supplementation were additionally excluded. This strict selection allowed us to evaluate the clinical effects of combining HM with diosmectite, which reflects a treatment approach commonly used in real-world practice. Furthermore, by simultaneously assessing both therapeutic efficacy and adverse events, this study provides a clinically meaningful evaluation of the benefit–risk profile of this combination therapy for pediatric rotavirus gastroenteritis.

This study has several limitations. First, although 12 databases were searched without language restrictions, all included RCTs were conducted in China. This geographical concentration may limit the generalizability of the findings to other regions, as differences in healthcare systems may restrict the direct applicability of the results. Second, substantial heterogeneity was observed across studies, which may be partly attributable to the wide variety of HM formulations used. Because HM is prescribed according to individualized pattern differentiation, it is difficult to apply a single standardized formulation to pediatric rotavirus gastroenteritis. Third, the findings should be interpreted with caution. HM was used as an adjunct therapy, and most trials incorporated standard supportive care. In addition, TER is inherently subjective, and its definitions vary across studies. Although reductions in inflammatory biomarkers (CRP, IL-6, and IL-8) were observed, these markers are not routinely assessed in uncomplicated pediatric rotavirus gastroenteritis, and their clinical relevance remains uncertain. Furthermore, while pooled analyses suggested fewer adverse events in the HM group, this does not confirm superior safety, particularly as many studies lacked systematic laboratory monitoring to detect potential hepatic or renal toxicity. Future studies should include standardized safety reporting, objective laboratory monitoring, and validated outcome measures to ensure a more reliable evaluation of HM efficacy and safety.

## 5. Conclusions

This systematic review and meta-analysis indicate that HM combined with diosmectite was associated with reductions in diarrhea duration and hospital stay, as well as fewer reported adverse events compared with diosmectite alone in children with rotavirus gastroenteritis. However, the overall certainty of evidence ranged from moderate to very low, and important methodological limitations, clinical heterogeneity of herbal formulations, and the regional concentration of included studies limit the strength and generalizability of these findings. Despite these limitations, these results indicate that HM in combination with diosmectite may be considered a potential adjunctive therapy alongside standard supportive treatment in the management of pediatric rotavirus gastroenteritis.

## Figures and Tables

**Table 1:** Basic characteristics of the included studies.

First Author (year)	Sample Size (E/C)	Gender (M/F)	Age Distribution (mean ± SD)	Duration of Disease (mean ± SD)	Experimental Intervention (E)	Total Treatment Periods	Outcome Measurement
					Control Intervention (C)		
Bai (2014) [16]	110 (60/50)	E: 60 (33/27) C: 50 (27/23)	E: (16 ± 4) m C: (16 ± 3) m	NR	(C) + HM	3 d	(1), (2), (5), (6)
					diosmectite + ST		
Chen (2025) [17]	100 (50/50)	E: 48 C: 47	E: (4.3 ± 3.17) yr C: (4.73 ± 2.55) yr	E: (1.12 ± 0.21) d C: (1.41 ± 0.39) d	(C) + HM	3 d	(1), (2), (5), (6), (8), (10), (11)
					diosmectite		
Cheng (2017) [18]	72 (36/36)	E: 36 (20/16) C: 36 (22/14)	E: (3.2 ± 0.5) yr C: (3.1 ± 0.4) yr	E: (2.3 ± 0.4) d C: (2.1 ± 0.3) d	(C) + HM	NR	(1), (2), (4), (5), (6)
					diosmectite + ST		
Chu (2006) [19]	85 (43/42)	E: 43 (30/15) C: 42 (29/13)	E: (2.3 ± 0.8) yr C: (2.1 ± 1.0) yr	E: (1.8 ± 1.1) d C: (1.5 ± 1.2) d	(C) + HM	3–5 d	(1), (2)
					diosmectite + ST		
Dai (2004) [20]	136 (68/68)	NR	7 m~2 yr	<3 d	(C) + HM	3 d	(2)
					diosmectite + ST		
Duan (2019) [21]	200 (100/100)	E: 100 (55/45) C: 100 (59/41)	E: (1.52 ± 0.79) yr C: (1.55 ± 1.08) yr	NR	(C) + HM	NR	(1), (2), (5), (6)
					diosmectite		
Gao (2006) [22]	97 (67/30)	E: 67 (37/30) C: 30 (15/15)	E: 2~12 m (n = 40) 13~24 m (n = 27) C: 2~12 m (n = 20) 13~24 m (n = 10)	E: <3 d (n = 45) >3 d (n = 22) C: <3 d (n = 19) >3 d (n = 11)	(C) + HM	NR	(2), (3)
					diosmectite + ST		
Hou (2004) [23]	160 (85/75)	E: 85 (56/29) C: 75 (50/25)	E: 5~12 m (n = 66) 13~24 m (n = 19) C: 5~12 m (n = 48) 13~24 m (n = 27)	NR	(C) + HM	<1 wk	(1), (2)
					diosmectite + ST		
Huang (2013) [24]	116 (58/58)	E: 30 (16/14) C: 30 (17/13)	(14.2 ± 10.1) m	NR	(C) + HM	3 d	(2)
					diosmectite + ST		
Kang (2013) [25]	94 (47/47)	E: 47 (31/16) C: 47 (30/17)	E: <2 yr (n = 40) >2 yr (n = 7) C: <2 yr (n = 38) >2 yr (n = 9)	E: (2.32 ± 0.8) d C: (2.50 ± 0.6) d	(C) + HM	7 d	(1), (2), (6), (9)
					diosmectite + ST		
Li (2007) [26]	98 (50/48)	E: 50 (23/27) C: 48 (22/26)	E: 5 m~3 yr C: 6 m~3.1 yr	<3 d	(C) + HM	NR	(2)
					diosmectite + ST		
Li (2019) [27]	130 (65/65)	E: 65 (35/30) C: 65 (36/29)	E: (1.28 ± 0.43) m C: (1.36 ± 0.38) m	E: (2.32 ± 0.41) d C: (2.25 ± 0.46) d	(C) + HM	5 d	(1), (2), (5), (6), (8)
					diosmectite + ST		
Liao (2012) [28]	174 (58/58/58)	174 (82/92)	(2.8 ± 1.78) yr	NR	(C) + HM	7 d	(2), (3)
					diosmectite + ST		
Liu (2005) [29]	97 (51/46)	NR	2 m~2 yr	NR	(C) + HM	NR	(1), (2), (3)
					diosmectite + ST		
Liu (2016) [30]	97 (49/48)	E: 49 (30/19) C: 48 (36/12)	E: 7~12 m (n = 8) 13~48 m (n = 41) C: 6~12 m (n = 13) 13~48 m (n = 35)	NR	(C) + HM	3 d	(2)
					diosmectite + ST		
Liu (2017) [31]	70 (35/35)	E: 35 (18/17) C: 35 (15/20)	E: 6 m~2 yr C: 7 m~3 yr	E: 3.5 d C: 3.4 d	(C) + HM	3 d	(1), (2), (5), (6)
					diosmectite		
Nie (2018) [32]	100 (50/50)	E: 50 (28/22) C: 50 (26/24)	E: (1.4 ± 0.3) yr C: (5.0 ± 1.0) yr	E: (1.15 ± 0.2) d C: (4.0 ± 0.5) d	(C) + HM	7 d	(2), (3), (14)
					diosmectite + ST		
Nie (2020) [33]	90 (45/45)	E: 45 (23/22) C: 45 (37/33)	E: 5 m~4 yr C: 7 m~3 yr	<3 d	(C) + HM	2 wk	(2)
					diosmectite		
Ran (2017) [34]	78 (39/39)	E: 39 (19/20) C: 39 (21/18)	E: (13.92 ± 10.58) m C: (14.28 ± 10.04) m	NR	(C) + HM	3 d	(1), (2), (12)
					diosmectite + ST		
Wu (2012) [35]	88 (56/32)	E: 56 (36/20) C: 32 (21/11)	E: 8~12 m (n = 10) 13~36 m (n = 46) C: 8~12 m (n = 4) 13~48 m (n = 28)	NR	(C) + HM	3 d	(2), (3)
					diosmectite + ST		
Xia (2014) [36]	312 (156/156)	E: 156 (81/75) C:156 (79/77)	E: (1.36 ± 0.32) yr C: (1.27 ± 0.23) yr	NR	(C) + HM	3 d	(2), (3), (4), (6), (11), (12), (15)
					diosmectite + ST		
Xie (2019) [37]	90 (45/45)	E: 45 (27/18) C: 45 (29/16)	E: (1.93 ± 0.75) yr C: (1.85 ± 0.92) yr	E: (1.68 ± 0.56) d C: (1.85 ± 0.92) d	(C) + HM	3 d	(1), (2), (5), (6), (10), (16)
					diosmectite + ST		
Xing (2014) [38]	64 (32/32)	E: 32 (17/15) C: 32 (15/17)	E: (10.6 ± 2.8) m C: (10.5 ± 2.6) m	E: (1.5 ± 0.5) d C: (1.5 ± 0.5) d	(C) + HM	3 d	(2)
					diosmectite + ST		
Yi (2018) [39]	80 (40/40)	E: 40 (22/18) C: 40 (19/21)	E: <12 m (n = 7) 13~36 m (n = 21) 37~84 m (n = 12) C: <12 m (n = 9) 13~36 m (n = 20) 37~84 m (n = 11)	NR	(C) + HM	5 d	(1), (2), (3), (4), (5), (12)
					diosmectite + ST		
Zhang (2009) [40]	68 (36/32)	E: 36 (20/16) C: 32 (21/11)	E: 6~12 m (n = 25) 13~24 m (n = 11) C: 6~12 m (n = 20) 13~24 m (n = 12)	NR	(C) + HM	3 d	(2)
					diosmectite + ST		
Zhang (2024) [41]	70 (35/35)	E: 35 (17/18) C: 35 (16/19)	E: (6.24 ± 1.59) yr C: (6.17 ± 1.43) yr	E: (3.55 ± 1.67) d C: (3.57 ± 1.42) d	(C) + HM	7 d	(1), (5), (6), (7), (8), (13)
					diosmectite		

E, experimental; C, control; SD, standard deviation; NR, not reported; d, day; wk, week; yr, year; HM, herbal medicine; ST, standard treatments; (1), duration of diarrhea; (2), total effective rate; (3), incidence of adverse events; (4), length of hospital stay (days); (5), time to cessation of vomiting (days); (6), time to cessation of fever (days); (7), time to cessation of abdominal pain (days); (8), changes in immune and inflammatory markers; (9), changes in serum creatine kinase and creatine kinase–MB levels; (10), stool frequency(days); (11), time to normalization of stool frequency (days); (12), time to normalization of stool consistency (days); (13), time to resolution of dehydration (days); (14), symptom scores; (15), treatment cost (Chinese yuan, CNY); (16), changes in gut microbiota composition.

**Table 2:** Herbal medicine information.

First Author (year)	Prescription/Composition	Dosage (Time)	Frequency (Day)
Bai (2014) [16]	Weichang an Pill (Tianjin Zhongxin Pharmaceutical Co., Ltd., Tian, China, Z10880010) Aucklandiae Radix, Aquilariae Lignum Resinatum, Santali Albi Lignum, Moschus, Cinnabaris, Aurantii Fructus, Magnoliae Officinalis Cortex, Chuanxiong Rhizoma, Rhei Radix et Rhizoma, Crotonis Fructus Pulveratum, and Jujubae Fructus	0–1 yr: 0.02 g 1–2 yr: 0.04 g	3 times
Chen (2025) [17]	Xiaoer Shikou Powder * (Ulanhot Zhongmeng Pharmaceutical Co., Ltd., Ulanhot, China, Z20090041)	2 g	3 time
Cheng (2017) [18]	Gegen Qinlian Decoction Puerariae Radix (9 g), Scutellariae Radix (3 g), Glycyrrhizae Radix et Rhizoma (3 g), and Coptidis Rhizoma (3 g)	NR	2 times
Chu (2006) [19]	Qiwei Baizhu Powder Codonopsis Radix (5 g), Astragali Radix (4 g), Atractylodis Macrocephalae Rhizoma (5 g), Poria (5 g), Puerariae Radix (6 g), Agastachis Herba (5 g), and Aucklandiae Radix (4 g)	50 mL/day	3 times
Dai (2004) [20]	Modified Yigong San Codonopsis Radix (3 g), Atractylodis Macrocephalae Rhizoma (fried, 3 g), Poria (3 g), Glycyrrhizae Radix et Rhizoma (honey-processed, 2 g), Citri Reticulatae Pericarpium (3 g), Puerariae Radix (processed, 3 g), Massa Medicata Fermentata (3 g), Crataegi Fructus (3 g), Hordei Fructus Germinatus (3 g), and Schisandrae Chinensis Fructus (2 g)	NR	NR
Duan (2019) [21]	Modified Gegen Qinlian Decoction Puerariae Radix (8 g), Coptidis Rhizoma (2 g), Scutellariae Radix (5 g), Glycyrrhizae Radix et Rhizoma (2 g), Agastachis Herba (5 g), Atractylodis Rhizoma (5 g), Amomi Fructus (3 g), Isatidis Radix (6 g), Aucklandiae Radix (3 g), Mume Fructus (6 g), Poria (10 g), Lophatheri Herba (3 g), and Zingiberis Rhizoma Recens (3 g)	NR	NR
Gao (2006) [22]	Xiaoer Zhixie Granules *	NR	NR
Hou (2004) [23]	Food retention–induced diarrhea: Baohe Pill Puerariae Radix (2 g), Scutellariae Radix (1.5 g), Coptidis Rhizoma (1.5 g), and Glycyrrhizae Radix et Rhizoma (1 g) Wind-cold diarrhea: Huoxiang Zhengqi Powder Agastachis Herba (3 g), Perillae Folium (2 g), Angelicae Dahuricae Radix (2 g), Arecae Pericarpium (2 g), Poria (3 g), Atractylodis Macrocephalae Rhizoma (3 g), Pinelliae Rhizoma Praeparatum cum Massa Medicata Fermentata (2 g), Magnoliae Officinalis Cortex (2 g), Platycodonis Radix (2 g), Zingiberis Rhizoma Recens (1 slice), and Jujubae Fructus (1 piece) Damp-heat diarrhea: Gegen Qinlian Decoction Puerariae Radix (2 g), Scutellariae Radix (1.5 g), Coptidis Rhizoma (1.5 g), and Glycyrrhizae Radix et Rhizoma (1 g) Spleen-deficiency diarrhea: Shenling Baizhu Powder Lablab Semen Album (2 g), Ginseng Radix (2 g), Atractylodis Macrocephalae Rhizoma (2 g), Poria (2 g), Glycyrrhizae Radix et Rhizoma (1.5 g), Dioscoreae Rhizoma (2 g), Nelumbinis Semen (2 g), Platycodonis Radix (1.5 g), Coicis Semen (2 g), and Amomi Fructus Rotundus (2 g) Spleen–kidney yang deficiency: warming and tonifying the spleen and kidney Aconiti Lateralis Radix Praeparata (1.5 g), Ginseng Radix (2 g), Atractylodis Macrocephalae Rhizoma (3 g), Zingiberis Rhizoma (2 g), Glycyrrhizae Radix et Rhizoma (1.5 g), Psoraleae Fructus (2 g), Schisandrae Chinensis Fructus (2 g), Myristicae Semen (2 g), Evodiae Fructus (2 g), Zingiberis Rhizoma Recens (1 slice), and Jujubae Fructus (1 piece)	5–10 mL	4 times
Huang (2013) [24]	Fengliao Changweikang Granules (Haikou Pharmaceutical Co., Ltd., Haikou, China, Z10910055) Strobilanthes cusia and Polygonum hydropiper	0–1 yr: 1 g 1–2 yr: 1.5 g >2 yr: 2 g	3 times
Kang (2013) [25]	Zhixie Decoction Poria (15 g), Codonopsis Radix (9 g), Dioscoreae Rhizoma (fried, 9 g), Puerariae Radix (20 g), Cinnamomi Ramulus (5 g), Alismatis Rhizoma (12 g), Hordei Fructus Germinatus (10 g), Massa Medicata Fermentata (10 g), Atractylodis Rhizoma (fried, 6 g), and Glycyrrhizae Radix et Rhizoma (6 g)	100 mL/day	3–4 times
Li (2007) [26]	Zhixie Weiling Decoction Atractylodis Rhizoma (1–2 g), Citri Reticulatae Pericarpium (1–2 g), Glycyrrhizae Radix et Rhizoma (1–2 g), Polyporus (3–6 g), Alismatis Rhizoma (3–6 g), Atractylodis Macrocephalae Rhizoma (3–6 g), Granati Pericarpium (3–6 g), Magnoliae Officinalis Cortex (3–6 g), Plantaginis Semen (6–9 g), and Poria (6–9 g)	NR	NR
Li (2019) [27]	Modified Gegen Qinlian Decoction Puerariae Radix (10 g), Glycyrrhizae Radix et Rhizoma (5 g), Scutellariae Radix (5 g), and Coptidis Rhizoma (5 g)	50 mL	2 times
Liao (2012) [28]	Zhenren Yangzang Decoction Ginseng Radix (5 g), Atractylodis Macrocephalae Rhizoma (fried, 6 g), Angelicae Sinensis Radix (3 g), Myristicae Semen (3 g), Cinnamomi Cortex (2 g), Glycyrrhizae Radix et Rhizoma (honey-processed, 3 g), Paeoniae Radix Alba (7 g), Aucklandiae Radix (2 g), Chebulae Fructus (6 g), and Papaveris Pericarpium (3 g)	75 mL	4 times
Liu (2005) [29]	Xiaoer Tuxiening Powder *	0–1 yr: 1/5–1/3 pack 1–3 yr: 1/3–1/2 pack 3–6 yr: 1/2–1 pack	3 times
Liu (2016) [30]	Erxieting Granules *	0–1 yr: 1 pack >1 yr: 2 pack	2 times
Liu (2017) [31]	Gegen Qinlian Decoction Pericarpium (3 g), Coptidis Rhizoma (3 g), Glycyrrhizae Radix et Rhizoma (3 g), Puerariae Radix (8 g), Poria (8 g), Coicis Semen (8 g), Hordei Fructus Germinatus (8 g), Galli Gigerii Endothelium Corneum (5 g), Massa Medicata Fermentata (5 g), Houttuyniae Herba (6 g), and Scutellariae Radix (6 g)	NR	NR
Nie (2018) [32]	Jianpi Qushi Decoction Atractylodis Rhizoma (fried, 6 g), Alismatis Rhizoma (6 g), Crataegi Fructus (6 g), Pseudostellariae Radix (8 g), Poria (8 g), Atractylodis Macrocephalae Rhizoma (8 g), Glycyrrhizae Radix et Rhizoma (3 g), and Citri Reticulatae Pericarpium (3 g)	0–1 yr: 30 mL/day >1 yr: 50 mL/day	3 times
Nie (2020) [33]	Zhixie Weiling Decoction Atractylodis Rhizoma (1–2 g), Citri Reticulatae Pericarpium (1–2 g), Glycyrrhizae Radix et Rhizoma (1–2 g), Polyporus (3–6 g), Alismatis Rhizoma (3–6 g), Atractylodis Macrocephalae Rhizoma (3–6 g), Granati Pericarpium (3–6 g), Magnoliae Officinalis Cortex (3–6 g), Plantaginis Semen (6–9 g), and Poria (6–9 g)	NR	2 times
Ran (2017) [34]	Fengliao Changweikang Granules Strobilanthes cusia and Polygonum hydropiper	0–1 yr: 1/3 pack 1–2 yr: 1/2 pack >2 yr: 1/3 pack	3 times
Wu (2012) [35]	Erxieting Granules (Hefei Shenlu Shuanghe Pharmaceutical Co., Ltd., Hefei, China, Z19990025)	0–1 yr: 1 g 1–2 yr: 2 g	3 times
Xia (2014) [36]	Compound Ocimum Oil Oral liquid Dingxiang Luole Oil (1.5 mg), Calcii Carbonas (50 mg), Aluminii Hydroxidum (16.7 mg), and Magnesii Trisilicas (11.5 mg)	0–1 yr: 5 mL 1–2 yr: 10 mL >2 yr: 20–40 mL	3 times
Xie (2019) [37]	Qiwei Baizhu Powder Agastachis Herba (10 g), Puerariae Radix (10 g), Atractylodis Macrocephalae Rhizoma (10 g), Poria (10 g), Codonopsis Radix (10 g), Aucklandiae Radix (4 g, added later), and Glycyrrhizae Radix et Rhizoma (3 g)	0–1 yr: 30 mL 1–2 yr: 40 mL 2–3 yr: 50 mL	3 times
Xing (2014) [38]	Shenling Baizhu Powder combined with Wuji Pill Coicis Semen (raw, 15 g), Amomi Fructus (3 g, added later), Glycyrrhizae Radix et Rhizoma (3 g), Poria (10 g), Codonopsis Radix (12 g), Atractylodis Macrocephalae Rhizoma (4 g), Platycodonis Radix (6 g), Dioscoreae Rhizoma (12 g), Coptidis Rhizoma (3 g), Evodiae Fructus (3 g), and Paeoniae Radix Alba (raw, 3 g)	NR	2 times
Yi (2018) [39]	Shuanghuanglian Oral Liquid (Henan Tailong Pharmaceutical Group Co., Ltd., Zhengzhou, China, Z20133010) Lonicerae Flos, Scutellariae Radix, Forsythiae Fructus	0–1 yr: 5 mL 1–3 yr: 10 mL 4–7 yr: 20 mL	3 times
Zhang (2009) [40]	Qiuxie Decoction Atractylodis Rhizoma (10 g), Poria (10 g), Polyporus (10 g), Polygonum chinense (10 g), Puerariae Radix (10 g), Crataegi Fructus Carbonisatus (10 g), Agastachis Herba (6 g), Magnoliae Officinalis Cortex (5 g), Citri Reticulatae Pericarpium (3 g), Saposhnikoviae Radix (3 g), and Glycyrrhizae Radix et Rhizoma (3 g)	adjusted based on age and body weight	2 times
Zhang (2024) [41]	Xingpi Yanger Granules * (Guizhou Jianxing Pharmaceutical Group Co., Ltd., Guiyang, China, Z20220115)	4 g	3 times

NR, not reported; yr, year; *, Detailed information on the herbal composition was not provided.

**Table 3:** The Quality of evidence.

Outcomes	No. Participants (Studies)	Anticipated Absolute Effects (95% CI)		Relative Effect (95% CI)	Heterogeneity (I^2^)	Quality of Evidence (GRADE)
		Risk with Control Group	Risk with Intervention Group			
Duration of diarrhea	1174 (12 RCTs)	-	SMD 1.31 lower (1.63 lower to 0.99 lower)	-	84	⊕◯◯◯ Very Low ^a,b,c^
Total effective rate	2743 (25 RCTs)	740 per 1000	925 per 1000 (970 to 881)	RR 1.25 (1.19 to 1.31)	53	⊕◯◯◯ Very Low ^a,b,c^
Incidence of adverse events	793 (6 RCTs)	27 per 1000	7 per 1000 (25 to 2)	RR 0.24 (0.06 to 0.92)	0	⊕⊕⊕◯ Moderate ^a^
CRP	165 (2 RCTs)	-	SMD 0.82 lower (1.13 lower to 0.5 lower)	-	35	⊕⊕⊕◯ Moderate ^a^
IL-6	295 (3 RCTs)	-	SMD 2.2 lower (4.22 lower to 0.18 lower)	-	98	⊕◯◯◯ Very Low ^a,b,d^
IL-8	165 (2 RCTs)	-	SMD 1.29 lower (1.63 lower to 0.96 lower)	-	0	⊕⊕⊕◯ Moderate ^a^
Length of hospital stay	464 (3 RCTs)	-	MD 1.53 lower (1.73 lower to 1.33 lower)	-	0	⊕⊕⊕◯ Moderate ^a^
Duration of vomiting	917 (9 RCTs)	-	SMD 1.4 lower (1.97 lower to 0.84 lower)	-	93	⊕⊕◯◯ Low ^a,b^
Duration of fever	931 (9 RCTs)	-	SMD 1.78 lower (2.48 lower to 1.07 lower)	-	95	⊕⊕◯◯ Low ^a,b^

^a^, Risk of bias (The overall bias is unclear in half or more of the studies); ^b^, Inconsistency (I^2^ > 50%, there are substantial heterogeneity); ^c^, Publication bias (Visual inspection of the funnel plot and Egger’s test indicated asymmetry); ^d^, Imprecision (very wide confidence interval); CI, confidence interval; RR, risk ratio; RCT, randomized controlled trial; SMD, standardized mean difference; GRADE, Grading of Recommendations Assessment, Development, and Evaluation; CRP, C-reactive protein; IL-6, Interleukin-6; IL-8, Interleukin-8; ⊕, Higher quality of evidence; ◯, Lower quality of evidence.

## Data Availability

All data analyzed in this study are available within this article and its Supplementary Materials.

## Supplementary Materials

The following supporting information can be downloaded at: https://www.mdpi.com/article/10.3390/healthcare14060711/s1, Supplementary Table S1: PRISMA 2020 checklist; Supplementary Table S2: Search strategy for each database; Supplementary Table S3: Frequency of herb; Supplementary Table S4: Diosmectite and Standard supportive treatments information; Supplementary Table S5: Outcome measurement & Result (*p*-value); Supplementary Table S6: Sensitivity analysis of TER.

## Abbreviations

The following abbreviations are used in this manuscript:

CI	confidence interval
CRP	C-reactive protein
GRADE	Grading of Recommendations Assessment, Development, and Evaluation
HM	herbal medicine
IL	interleukin
MD	mean difference
RCTs	randomized clinical trials
RoB	risk of bias
RR	risk ratio
SMD	standardized mean difference
TER	total effective rate
