# Supplementary material for: Efficacy and Safety of Oral Herbal Medicine Combined with Diosmectite for Pediatric Rotavirus Gastroenteritis: A Systematic Review and Meta-Analysis
Source: Healthcare (Basel). 2026 Mar 11;14(6):711. doi: 10.3390/healthcare14060711 (PMC13026062; doi:10.3390/healthcare14060711)
Supplement: Supplementary file 1 [file healthcare-14-00711-s001.zip › Supplementary Table S2. Search strategy used in each database_Rota.pdf]

Supplementary Table S2. Search strategy used in each database.

Medline via PubMed

	Searches	Results
#1	"Infant"[MeSH Terms] OR "Child"[MeSH Terms] OR "Pediatrics"[MeSH Terms] OR "infant*"[Title/Abstract] OR "child*"[Title/Abstract] OR "pediatric*"[Title/Abstract]	***3,702,824***
#2	("Rotavirus"[MeSH Terms] OR "rotavirus*"[Title/Abstract]) AND ("Enteritis"[MeSH Terms] OR "Enteritis"[Title/Abstract] OR "Diarrhea"[MeSH Terms] OR "diarrhea*"[Title/Abstract])	***7458***
#3	"drugs, chinese herbal"[MeSH Terms] OR "medicine, chinese traditional"[MeSH Terms] OR "medicine, kampo"[MeSH Terms] OR "medicine, korean traditional"[MeSH Terms] OR "Korean medicine"[Title/Abstract] OR "Chinese medicine"[Title/Abstract] OR "Oriental medicine"[Title/Abstract] OR "Kampo medicine"[Title/Abstract] OR "herbal medicine"[Title/Abstract] OR "herb*"[Title/Abstract] OR "formul*"[Title/Abstract] OR "remed*"[Title/Abstract] OR "plant*"[Title/Abstract] OR "decoction*"[Title/Abstract] OR "solution*"[Title/Abstract] OR "liquid*"[Title/Abstract] OR "extract*"[Title/Abstract] OR "capsule*"[Title/Abstract] OR "powder*"[Title/Abstract]	***3,977,044***
#4	#1 AND #2 AND #3	***509***
#5	Filters: Randomized Controlled Trial	***72***

EMBASE

	Searches	Results
#1	'infant'/exp OR 'child'/exp OR 'pediatrics'/exp OR infant*:ti,ab OR child*:ti,ab OR pediatric*:ti,ab	***4,748,884***
#2	'rotavirus'/exp OR rotavirus*:ti,ab	***781,671***
#3	'enteritis'/exp OR enteritis:ti,ab OR 'diarrhea'/exp OR diarrhea*:ti,ab	***4,834,922***
#4	'chinese herbal drug' OR 'traditional chinese medicine'/exp OR 'kampo medicine'/exp OR 'traditional korean medicine'/exp OR 'korean medicine':ti,ab OR 'chinese medicine':ti,ab OR 'oriental medicine':ti,ab OR 'kampo medicine':ti,ab OR 'herbal medicine':ti,ab OR herb*:ti,ab OR formul*:ti,ab OR remed*:ti,ab OR plant*:ti,ab OR decoction*:ti,ab OR solution*:ti,ab OR liquid*:ti,ab OR extract*:ti,ab OR capsule*:ti,ab OR powder*:ti,ab	***2,610,091***
#5	#1 AND #2 AND #3 AND #4	***973***
#6	#5 AND 'randomized controlled trial'/de	***130***

CENTRAL

	Searches	Results
#1	MeSH descriptor: [Rotavirus] explode all trees	***291***
#2	MeSH descriptor: [Enteritis] explode all trees	***307***
#3	MeSH descriptor: [Diarrhea] explode all trees	***4451***
#4	#1 AND (#2 OR #3)	***92***
#5	"rotavirus gastroenteritis" OR "autumn diarrhea"	***181***
#6	#4 OR #5	***206***
#7	MeSH descriptor: [Pediatrics] explode all trees	***1075***
#8	MeSH descriptor: [Infant] explode all trees	***46560***
#9	MeSH descriptor: [Child] explode all trees	***84008***
#10	pediatric OR child* OR Infant	***281712***
#11	#7 OR #8 OR #9 OR #10	***281759***
#12	MeSH descriptor: [Herbal Medicine] explode all trees	***96***
#13	MeSH descriptor: [Medicine, Chinese Traditional] explode all trees	***1877***
#14	MeSH descriptor: [Medicine, Kampo] explode all trees	***66***

#15	Korean medicine OR Chinese medicine OR Oriental medicine OR Kampo medicine OR herbal medicine OR herb* OR formul* OR remed* OR plant* OR decoction* OR solution* OR liquid* OR extract* OR capsule* OR powder*	***316887***
#16	#12 OR #13 OR #14 OR #15	***317023***

CNKI

#1	SU=('pediatric'+'children'+'儿童'+'小儿') AND SU=('rotavirus enteritis'+'秋季腹泻'+'rotavirus diarrhea'+'autumn diarrhea') AND SU=('Chinese herbal drugs'+'traditional Chinese medicine' +'Chinese herbal medicine'+'traditional medicine'+'decoction'+'extract'+'oil' +'herbal' + 'herb'+'中药' +'中医药'+'中成药'+'汤'+'丸'+'散'+'方'+'颗粒'+'胶囊'+'自拟')	***1516***

Wan Fang Database

#1	主题:(pediatric or children or 儿童 or 小儿) and 主题:(rotavirus enteritis or 秋季腹泻 ro rotavirus diarrhea ro autumn diarhea) and 主题:(Chinese herbal drugs or traditional Chinese medicine or Chinese herbal medicine or traditional medicine or decoction or extract or oil or herbal or herb or 中药 or 中医药 or 中成药 or 汤 or 丸 or 散 or 方 or 颗粒 or 胶囊 or 自拟)	***812***

Chinese Scientific Journal Database (VIP)

#1	M=(pediatric OR children OR 儿童 OR 小儿) AND M=(rotavirus enteritis OR 秋季腹泻 OR rotavirus diarrhea OR autumn diarrhea) AND M=(Chinese herbal drugs OR traditional Chinese medicine OR Chinese herbal medicine OR traditional medicine OR decoction OR extract OR oil OR herbal OR herb OR 中药 OR 中医药 OR 汤 OR 丸 OR 散 OR 方 OR 颗粒 OR 胶囊 OR 自拟)	***737***

Oriental Medicine Advanced Searching Integrated System (OASIS)

#1	(설사 OR 장염 OR 로타바이러스) AND 한약	***4***

Korean studies Information Service System (KISS)

#1	(설사 OR 장염 OR 로타바이러스) AND 한약	***0***

Korea Citation Index (KCI)

#1	(설사 OR 장염 OR 로타바이러스) AND 한약	***7***

Research Information Sharing Service (RISS)

#1	(설사 OR 장염 OR 로타바이러스) AND 한약	***20***

Korean Medical database (KMbase)

#1	(((설사|abstract) OR (장염|abstract)) OR (로타바이러스|abstract)) AND (한약|abstract)	***8***

Citation Information by NI (CiNii)

#1	("rotavirus enteritis" OR "rotavirus diarrhea" OR "autumn diarrhea" OR diarrhea OR 下痢 OR ロタウイルス) AND ("traditional Korean medicine" OR "traditional Chinese medicine" OR "traditional oriental medicine" OR "Kampo medicine" OR "alternative medicine" OR "complementary medicine" OR herb OR herbs OR herbal OR decoction OR botanical OR 漢方 OR 漢方薬 OR 生薬 OR 湯 OR 散 OR 丸) AND ("randomized controlled trial" OR randomized OR randomised OR RCT OR "clinical trial" OR 無作為化 OR ランダム化 OR 無作為化比較試験 OR ランダム化比較試験)	***48***
