# Supplementary material for: Efficacy and Safety of Oral Herbal Medicine Combined with Diosmectite for Pediatric Rotavirus Gastroenteritis: A Systematic Review and Meta-Analysis
Source: Healthcare (Basel). 2026 Mar 11;14(6):711. doi: 10.3390/healthcare14060711 (PMC13026062; doi:10.3390/healthcare14060711)
Supplement: Supplementary file 1 [file healthcare-14-00711-s001.zip › Supplementary Table S3. Frequency of herb_Rota.pdf]

Supplementary Table S3. Frequency of herb.

Frequency	Herb
17	Glycyrrhizae Radix et Rhizoma
13	Poria
11	Puerariae Radix, Atractylodis Macrocephalae Rhizoma
7	Coptidis Rhizoma, Scutellariae Radix
6	Citri Reticulatae Pericarpium, Atractylodis Rhizoma
5	Aucklandiae Radix, Magnoliae Officinalis Cortex, Codonopsis Radix, Agastachis Herba
4	Alismatis Rhizoma
4	Scorpio, Pinelliae Tuber, Lycii Radicis Cortex
3	Jujubae Fructus, Massa Medicata Fermentata, Hordei Fructus Germinatus, Zingiberis Rhizoma Recens, Platycodonis Radix, Ginseng Radix, Dioscoreae Rhizoma
