# Supplementary material for: Efficacy and Safety of Oral Herbal Medicine Combined with Diosmectite for Pediatric Rotavirus Gastroenteritis: A Systematic Review and Meta-Analysis
Source: Healthcare (Basel). 2026 Mar 11;14(6):711. doi: 10.3390/healthcare14060711 (PMC13026062; doi:10.3390/healthcare14060711)
Supplement: Supplementary file 1 [file healthcare-14-00711-s001.zip › Supplementary Table S4. Diosmectite and Standard supportive treatments information_Rota.pdf]

Supplementary Table S4. Diosmectite and Standard supportive treatments information

First author (year)	Diosmectite preparations	Dosage(time) / Frequency(day)	Standard supportive treatments
Bai (2014) [16]	montmorillonite powder	<1 y, 1.0 g<br>1-2 y, 1.5 g<br>3 times	(1)Fluid therapy for the prevention and correction of dehydration and acidosis<br>(2)Symptomatic management
Chen (2025) [17]	montmorillonite powder	3 g<br>3 times	NR
Cheng (2017) [18]	Smecta	<1 y, 1 g<br>1-2 y, 1-2 g<br>> 2 y, 2-3 g<br>3 times	(1)Fluid therapy for the prevention and correction of dehydration and acidosis
Chu (2006) [19]	Smecta	<1 y, 1 g<br>1-3 y, 1.5 g<br>3 times	(1)Fluid therapy for the prevention and correction of dehydration and acidosis
Dai (2004) [20]	Smecta	NR<br>NR	(1)Fluid therapy for the prevention and correction of dehydration and acidosis
Duan (2019) [21]	Smecta	<1 y, 1 g<br>1-3 y, 1.5 g<br>> 3 y, 3 g<br>3 times	NR
Gao (2006) [22]	Smecta	NR<br>NR	(1)Fluid therapy for the prevention and correction of dehydration and acidosis
Hou (2004) [23]	Smecta	6 m-1 y, 1 g<br>1-2 y, 1.5 g<br>3 times	(1)Oral rehydration salts (ORS) for the prevention of dehydration<br>(2)Dietary guidance
Huang (2013) [24]	montmorillonite powder	<1 y, 1 g<br>1-2 y, 1.5 g<br>> 2 y, 2 g<br>3 times	(1)Oral rehydration salts (ORS) or intravenous fluids for the prevention and correction of dehydration and acidosis<br>(2)Dietary guidance
Kang (2013) [25]	Smecta	<1 y, 1-1.5 g<br>1-2 y, 1.5-2 g<br>3 times	(1) Fluid therapy
Li (2007) [26]	montmorillonite powder	<1 y, 1 g<br>1-2 y, 1-2 g	(1)Oral rehydration salts (ORS) or intravenous fluids for the prevention and correction of dehydration

		>2 y, 2-3 g<br>3 times	
Li (2019) [27]	montmorillonite powder	<1 y, 1 g<br>1-3 y, 1.5 g<br>>3 y, 3 g<br>3 times	(1)Fluid therapy for the prevention and correction of dehydration and acidosis
Liao (2012) [28]	montmorillonite powder	6 g<br>3 times	(1)Fluid therapy for the prevention and correction of dehydration and acidosis
Liu (2005) [29]	Smecta	<1 y, 1 g<br>1-2 y, 1-2 g<br>3 times	(1)Oral rehydration salts (ORS) or intravenous fluids for the prevention and correction of dehydration and acidosis<br>(2)Dietary guidance
Liu (2016) [30]	Smecta	<1 y, 1 g<br>1-3 y, 2 g<br>>3 y, 3 g<br>3 times	(1)Fluid therapy for the prevention and correction of dehydration and acidosis
Liu (2017) [31]	montmorillonite powder	6 m-1 y, 1 g<br>1-2 y, 2 g<br>2-3 y, 3 g<br>3 times	NR
Nie (2018) [32]	Smecta	<1 y, 1 g<br>1-2 y, 1.5 g<br>3 times	(1)Fluid therapy for the prevention and correction of dehydration and acidosis<br>(2)Symptomatic management
Nie (2020) [33]	montmorillonite powder	<1 y, 1 g<br>1-2 y, 2 g<br>>2 y, 3 g<br>3 times	NR
Ran (2017) [34]	montmorillonite powder	<1 y, 1 g<br>1-2 y, 2 g<br>>2 y, 3 g<br>3 times	(1)Fluid therapy for the prevention and correction of dehydration and acidosis<br>(2)Dietary guidance
Wu (2012) [35]	Smecta	1-3 g<br>3 times	(1)Oral rehydration salts (ORS) or intravenous fluids for the prevention and correction of dehydration and acidosis
Xia (2014) [36]	montmorillonite powder	<1 y, 1 g<br>1-2 y, 1-2 g<br>>2 y, 2-3 g	(1)Intravenous fluids for the prevention and correction of dehydration and acidosis<br>(2)Symptomatic management

		3 times	
Xie (2019) [37]	Smecta	6 m-1 y, 1 g<br>1-2 y, 1.5 g<br>2-3 y, 2 g<br>3 times	(1)Oral rehydration salts (ORS) or intravenous fluids for the prevention and correction of dehydration and acidosis<br>(2)Dietary guidance
Xing (2014) [38]	Smecta	<1 y, 1 g<br>1-2 y, 2 g<br>>2 y, 3 g<br>3 times	(1)Oral rehydration salts (ORS) or intravenous fluids for the prevention and correction of dehydration and acidosis<br>(2)Symptomatic management
Yi (2018) [39]	montmorillonite powder	<1 y, 1 g<br>1-2 y, 1-2 g<br>>2 y, 2-3 g<br>3 times	(1)Oral rehydration salts (ORS) for the prevention and correction of dehydration and acidosis<br>(2)Dietary guidance
Zhang (2009) [40]	Smecta	<1 y, 1 g<br>1-2 y, 2 g<br>3 times	(1)Fluid therapy for the prevention and correction of dehydration and acidosis<br>(2)Dietary guidance
Zhang (2024) [41]	montmorillonite powder	3 g<br>3 times	NR
