# Supplementary material for: Efficacy and Safety of Oral Herbal Medicine Combined with Diosmectite for Pediatric Rotavirus Gastroenteritis: A Systematic Review and Meta-Analysis
Source: Healthcare (Basel). 2026 Mar 11;14(6):711. doi: 10.3390/healthcare14060711 (PMC13026062; doi:10.3390/healthcare14060711)
Supplement: Supplementary file 1 [file healthcare-14-00711-s001.zip › Supplementary Table S5. Outcome measurement & Result (P-value)_Rota.pdf]

Supplementary Table S5. Outcome measurement & Result (p -value).

First author (year)	Outcome measurement	Result (p -value)
Bai (2014) [16]	(1)Duration of diarrhea<br>(2)Total effective rate<br>(3)Time to cessation of vomiting (days)<br>(4)Time to cessation of fever (days)	(1) E < C[a]<br>(2) E > C[a]<br>(3) E < C[b]<br>(4) E < C[b]
Chen (2025) [17]	(1)Duration of diarrhea<br>(2)Total effective rate<br>(3)Time to cessation of vomiting (days)<br>(4)Time to cessation of fever (days)<br>(5)Changes in immune and inflammatory markers: CRP(mg/L), IL-6(pg/mL), IL-8(pg/mL)<br>(6)Stool frequency(days)<br>(7)Time to normalization of stool frequency (days)	(1) E < C[a]<br>(2) E > C[a]<br>(3) E < C[a]<br>(4) E < C[a]<br>(5) E < C[a]<br>(6) E < C[a]<br>(7) E < C[a]
Cheng (2017) [18]	(1)Duration of diarrhea<br>(2)Total effective rate<br>(3)Length of hospital stay (days)<br>(4)Time to cessation of vomiting (days)<br>(5)Time to cessation of fever (days)	(1) E < C[a]<br>(2) E > C[a]<br>(3) E < C[a]<br>(4) E < C[a]<br>(5) E < C[a]
Chu (2006) [19]	(1)Duration of diarrhea<br>(2)Total effective rate	(1) E < C[b]<br>(2) E > C[b]
Dai (2004) [20]	(1) Total effective rate	(1) E > C[a]
Duan (2019) [21]	(1)Duration of diarrhea<br>(2)Total effective rate<br>(3)Time to cessation of vomiting (days)<br>(4)Time to cessation of fever (days)	(1) E < C[b]<br>(2) E > C[a]<br>(3) E < C[a]<br>(4) E < C[a]
Gao (2006) [22]	(1)Total effective rate<br>(2)Incidence of adverse events	(1) E > C[b]<br>(2) No adverse events
Hou (2004) [23]	(1)Duration of diarrhea<br>(2)Total effective rate	(1) E < C[b]<br>(2) E > C[b]
Huang (2013) [24]	(1)Total effective rate	(1) E > C[a]

Kang (2013) [25]	(1)Duration of diarrhea<br>(2)Total effective rate<br>(3)Time to cessation of fever (hours)<br>(4)Changes in serum creatine kinase levels<br>(5) Changes in serum creatine kinase–MB levels	(1) E < C[a]<br>(2) E > C[a]<br>(3) E < C[b]<br>(4) E < C[b]<br>(5) E < C[a]
Li (2007) [26]	(1)Total effective rate	(1) E > C[b]
Li (2019) [27]	(1)Duration of diarrhea<br>(2)Total effective rate<br>(3)Time to cessation of vomiting (days)<br>(4)Time to cessation of fever (days)<br>(5)Changes in immune and inflammatory markers: IL-6(pg/mL), TNF-α(mg/L)	(1) E < C[a]<br>(2) E > C[a]<br>(3) E < C[a]<br>(4) E < C[a]<br>(5) E < C[a]
Liao (2012) [28]	(1)Total effective rate<br>(2)Incidence of adverse events	(1) E > C[a]<br>(2) E < C*: 6 cases vs 2 cases
Liu (2005) [29]	(1)Duration of diarrhea<br>(2)Total effective rate<br>(3)Incidence of adverse events	(1) E < C*<br>(2) E > C*<br>(3) E < C: No adverse events vs abdominal distension and constipation (number of cases was not reported)
Liu (2016) [30]	(1)Total effective rate	(1) E > C[a]
Liu (2017) [31]	(1)Duration of diarrhea<br>(2)Total effective rate<br>(3)Time to cessation of vomiting (hours)<br>(4)Time to cessation of fever (hours)	(1) E < C[a]<br>(2) E > C[a]<br>(3) E < C[a]<br>(4) E < C[a]
Nie (2018) [32]	(1)Total effective rate<br>(2)Incidence of adverse events<br>(3)Symptom scores	(1) E > C[b]<br>(2) E < C[a]: 0 cases vs 6 cases (dry stool (n = 4) and constipation (n = 2))<br>(3) E < C[b]
Nie (2020) [33]	(1)Total effective rate	(1) E > C[b]
Ran (2017) [34]	(1)Duration of diarrhea<br>(2)Total effective rate<br>(3)Time to normalization of stool consistency (days)	(1) E < C[a]<br>(2) E > C[a]<br>(3) E < C[a]
Wu (2012) [35]	(1)Total effective rate<br>(2)Incidence of adverse events	(1) E > C[a]<br>(2) No adverse events
Xia (2014) [36]	(1)Total effective rate<br>(2)Incidence of adverse events<br>(3)Length of hospital stay (days)<br>(4)Time to cessation of fever (days)	(1) E > C[a]<br>(2) No adverse events*<br>(3) E < C[a]<br>(4) E < C[a]

	(5)Time to normalization of stool frequency (days)<br>(6)Time to normalization of stool consistency (days)<br>(7)Treatment cost (Chinese yuan, CNY)	(5) E < C[a]<br>(6) E < C[a]<br>(7) E < C[a]
Xie (2019) [37]	(1)Duration of diarrhea<br>(2)Total effective rate<br>(3)Time to cessation of vomiting (days)<br>(4)Time to cessation of fever (days)<br>(5)Stool frequency(days)<br>(6)Changes in gut microbiota composition<br>1)*Bifidobacterium* and *Lactobacillus*<br>2)*Escherichia coli*	(1) E < C[b]<br>(2) E > C[a]<br>(3) E < C[b]<br>(4) E < C[b]<br>(5) E < C[a]<br>(6)<br>1) E > C[a]<br>2) E < C[a]
Xing (2014) [38]	(1)Total effective rate	(1) E > C[a]
Yi (2018) [39]	(1)Duration of diarrhea<br>(2)Total effective rate<br>(3)Incidence of adverse events<br>(4)Length of hospital stay (days)<br>(5)Time to cessation of vomiting (days)<br>(6)Time to normalization of stool consistency (days)	(1) E < C[a]<br>(2) E > C[a]<br>(3) No adverse events<br>(4) E < C*<br>(5) E < C[a]<br>(6) E < C[a]
Zhang (2009) [40]	(1)Total effective rate:<br>1)overall efficacy<br>2)vomiting<br>3)fever	(1)<br>1) E > C[b]<br>2) E > C[a]<br>3) E > C[a]
Zhang (2024) [41]	(1)Duration of diarrhea<br>(2)Time to cessation of vomiting (days)<br>(3)Time to cessation of fever (days)<br>(4)Time to cessation of abdominal pain (days)<br>(5)Changes in immune and inflammatory markers: CRP(mg/L), IL-6(pg/mL), IL-8(pg/mL)<br>(6)Time to resolution of dehydration (days)	(1) E < C[b]<br>(2) E < C[b]<br>(3) E < C[b]<br>(4) E < C[b]<br>(5) E < C[b]<br>(6) E < C[b]

a, $P<0.05$; b, $P<0.01$; *, not reported; E, experimental; C, control; CRP, C-reactive protein; IL-6, Interleukin-6; IL-8, Interleukin-8; TNF-α, Tumor necrosis factor-alpha
