# Supplementary material for: Efficacy and Safety of Oral Herbal Medicine Combined with Diosmectite for Pediatric Rotavirus Gastroenteritis: A Systematic Review and Meta-Analysis
Source: Healthcare (Basel). 2026 Mar 11;14(6):711. doi: 10.3390/healthcare14060711 (PMC13026062; doi:10.3390/healthcare14060711)
Supplement: Supplementary file 1 [file healthcare-14-00711-s001.zip › Supplementary Table S6. Sensitivity analysis for TER_Rota.pdf]

**Supplementary Table S6.** Sensitivity analysis for total effective rate.

| Excluding study | RR | 95%CI | $I^2$ (%) | *p*-value |
|---|---|---|---|---|
| Bai (2014) [16] | 1.25 | 1.19,1.32 | 55 | <0.00001 |
| Chen (2025) [17] | 1.25 | 1.19,1.32 | 56 | <0.00001 |
| Cheng (2017) [18] | 1.25 | 1.19,1.32 | 55 | <0.00001 |
| Chu (2006) [19] | 1.25 | 1.19,1.32 | 55 | <0.00001 |
| Dai (2004) [20] | 1.24 | 1.18,1.31 | 52 | <0.00001 |
| Duan (2019) [21] | 1.26 | 1.20,1.33 | 53 | <0.00001 |
| Gao (2006) [22] | 1.25 | 1.19,1.32 | 55 | <0.00001 |
| Hou (2004) [23] | 1.26 | 1.20,1.32 | 48 | <0.00001 |
| Huang (2013) [24] | 1.26 | 1.19,1.32 | 55 | <0.00001 |
| Kang (2013) [25] | 1.26 | 1.19,1.32 | 56 | <0.00001 |
| Li (2007) [26] | 1.25 | 1.19,1.31 | 55 | <0.00001 |
| Li (2019) [27] | 1.26 | 1.19,1.32 | 55 | <0.00001 |
| Liao (2012) [28] | 1.24 | 1.18,1.30 | 48 | <0.00001 |
| Liu (2005) [29] | 1.23 | 1.18,1.28 | 34 | <0.00001 |
| Liu (2016) [30] | 1.26 | 1.20,1.33 | 51 | <0.00001 |
| Liu (2017) [31] | 1.25 | 1.19,1.32 | 55 | <0.00001 |
| Nie (2018) [32] | 1.26 | 1.19,1.32 | 55 | <0.00001 |
| Nie (2020) [33] | 1.24 | 1.18,1.30 | 51 | <0.00001 |
| Ran (2017) [34] | 1.26 | 1.19,1.32 | 55 | <0.00001 |
| Wu (2012) [35] | 1.26 | 1.19,1.32 | 55 | <0.00001 |
| Xia (2014) [36] | 1.26 | 1.19,1.33 | 55 | <0.00001 |
| Xie (2019) [37] | 1.25 | 1.19,1.32 | 56 | <0.00001 |
| Xing (2014) [38] | 1.25 | 1.19,1.32 | 55 | <0.00001 |
| Yi (2018) [39] | 1.25 | 1.19,1.32 | 55 | <0.00001 |
| Zhang (2009) [40] | 1.24 | 1.18,1.30 | 50 | <0.00001 |

RR, risk ratio; CI, confidence interval.
